# Functional, nutritional, and sensorial evaluation of sorghum‐based beverages produced by single‐ and two‐stage acid, α‐amylase enzyme, and germination treatments

**DOI:** 10.1002/fsn3.4299

**Published:** 2024-08-22

**Authors:** Seyed Hasan Sajjadi Alhashem, Mohammad Reza Ehsani, Afshin Akhondzadeh Basti, Anoosheh Sharifan

**Affiliations:** ^1^ Department of Food Science and Technology, Science and Research Branch Islamic Azad University Tehran Iran; ^2^ Department of Food Hygiene, Faculty of Veterinary Medicine University of Tehran Tehran Iran

**Keywords:** acid treatments, functional food, nutritional compounds, plant‐based beverage, sprouting treatments, α‐Amylase treatments

## Abstract

Nowadays, the consumption of functional foods, such as plant‐based beverages, is increasing due to their health‐promoting properties. The low extraction yield of nutritional and functional components is considered a major challenge during the production of sorghum‐based beverages (SBB), as well as their sensorial properties. This investigation studied the effects of various treatments (acidic using phosphoric acid, enzymatic using α‐amylase, germination, germination‐acidic, germination‐enzymatic, and acidic‐enzymatic) on the functional, nutritional, and sensorial properties of SBB. The two‐stage acidic‐enzymatic treatment demonstrated the highest extraction yield, dry matter, ash, carbohydrates, and reducing sugar contents, as well as the lowest starch content (*p* < .05). Furthermore, the highest protein content (0.98%) was achieved by the germination treatments of sorghum grains. While the highest fat content was achieved by the acidic treatment (1.38%), the germination‐acidic treatment exhibited the highest energy value (26.02 kcal/100 mL). Moreover, the total phenolic content of the acidic‐enzymatic treatment (44.56 mg GAE/L) was significantly higher than that of other treatments. However, all treatments demonstrated lower antioxidant properties compared to the control treatment (142.85 mg BHT eq./L). Furthermore, the sensory evaluation of the germination and germination‐enzymatic treatments showed acceptable scores (≥7) for consumers. In conclusion, the results indicated that the two‐stage treatments of sorghum, especially the acidic‐enzymatic treatment, were more effective than single treatments for the extraction of functional and nutritional components during the production of SBB.

## Graphical abstract

Sorghum beverages produced by phosphoric acid, germination, α‐amylase, and two‐stage combinations of those showed higher beverage and protein extraction yields. All treatments showed higher total phenolic content but lower antioxidant activity. The consumer acceptability of all tretaments increased.

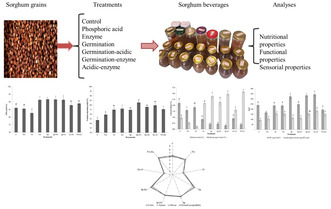

## 1 INTRODUCTION

Functional foods are foods or food ingredients that provide health benefits beyond their nutritional value (Charalampopoulos et al., 2002). *Sorghum bicolor* (L.) is a drought‐tolerant crop that is cultivated in semi‐arid regions of various countries. Sorghum is one of the major cereal grains and contains high levels of diverse and unique bioactive components, such as phenolic acids, flavonoid derivatives, and bioactive lipids with functional properties, located in the bran fraction (Althwab et al., 2015; Awika & Rooney, 2004; Charalampopoulos et al., 2002; De Morais Cardoso et al., 2017). Moreover, sorghum endosperm contains starch with lower digestibility, which has health benefits (Simnadis et al., 2016).

Despite their diverse nutritional components, sorghum grains (SG) face various challenges to becoming a novel human food source. In addition to the technological challenges, such as the lack of unique milling systems, no exclusive treatments have been developed for SG. The technological and biological availability of the nutritional components of sorghum is reduced due to their entrapment or binding by the hydrophobic protein matrix, the cell wall materials, and the presence of tannins (Taylor & Emmambux, 2010).

The global consumption of different plant‐based beverages is increasing (Sethi et al., 2016), and researchers encounter challenges that affect the functional, rheological, and sensorial properties of the products (Babolanimogadam et al., 2023). The composition of plant‐based beverages, which are produced by breaking down and extracting plant materials, depends on the plant source and the type of treatment (Babolanimogadam et al., 2023; Kumar et al., 2020). The main challenges for the production of sorghum‐based beverages (SBB) are to increase the extraction yield of different components, such as proteins, phenolics, and other functional compounds.

In this study, we aim to produce SBB with different treatments, such as acidic, enzymatic, germination alone, and in combination, to develop a high‐yield functional beverage.

## 2 MATERIALS AND METHODS

### 2.1 Materials

The SG (IGS; Fuman type KGS23) were purchased from Karaj Seed and Plant Improvement Institute (Alborz, Iran). The α‐amylase of *Aspergillus oryzae* was obtained from DSM Nutritionals (11,500 fungal amylase units (FAU)/g, Heerlen, The Netherlands). Double‐distilled water was used in all experiments. Other chemicals were purchased from Merck KGaA (Darmstadt, Germany).

### 2.2 Preparation of the grains

The grains were cleaned and disinfected with NaClO (0.1%) in a ratio of 1:6 (w/v) for 45 min and rinsed six times with distilled water at 5‐min intervals (Elkhalifa & Bernhardt, 2013). The grains were then dried, except for those used for germination. For the germination of SG, the traditional Sudanese method of germination was followed (Bhise et al., 1988). The SG were soaked for 48 h in sterile water with a ratio of 1:6 (w/v) at 25°C. The grains were stored at a higher than 90% relative humidity condition by keeping them under a moist sterile cloth in the dark for 96 h and placing the soaked seeds on wet filter paper and on a metal mesh under which water was placed in a sterile plastic container. Finally, all the grains were dried at 45°C until the moisture content reached about 5%. All SG were milled to obtain a particle size smaller than 0.5 mm and stored at 4°C until use.

### 2.3 Preparation of the treatments

Treatments (*n* = 7) were prepared by different treatments, described below. All treatments were prepared by adding 500 mL of water to 100 g of milled grains. The control treatment (CT) and germination treatment (GT) were prepared by heating the milled grains at 85°C for 15 min. For the preparation of acid treatments (AT), 5 mL of phosphoric acid (85%) was added to the slurry (pH = 2) and heated at 80°C for 60 min. For the preparation of enzyme treatment (En), the sorghum slurry was heated at 85°C for 15 min and cooled to 65°C. Then the alpha‐amylase enzyme (200 mg) was added to the slurry and incubated at 65°C for 60 min. Finally, the slurry was cooled to 25°C in an ice bath. For the preparation of acid‐enzymatic treatment (AET), the pH of the AT slurry was adjusted to 6.7 (by using NaOH; 20% w/v). Then, the slurry was prepared as En. The germination‐acidic treatment (GAT) and germination‐enzymatic treatment (GET) were prepared by using germinated slurry and five mL of phosphoric acid and treated as AT and En, respectively.

### 2.4 Production of SBB

Subsequent to the treatment applications, the pH values of the slurries exhibited a decrease. To normalize these values, the pH of each slurry was meticulously adjusted to 6.7. This was accomplished through the careful titration of a sodium hydroxide solution (NaOH; 20% w/v), with the pH readings being precisely monitored via a calibrated pH meter (Model PB‐11, Sartorius, Germany). To ensure an even and consistent distribution of NaOH within the slurry, a magnetic stirrer was employed throughout the adjustment process. Finally, the weight of the mixture was adjusted to 1000 g by using water. All slurries were manually filtered using 50‐grade cheesecloth. The yield of SBB was measured, and the samples were heated at 85°C for 15 min. The thermal adequacy was confirmed by culturing the SBB samples on BHI agar and incubating at 37°C for 72 h.

### 2.5 Proximate composition analysis

A pH meter (PB‐11, Sartorius, Germany) was used to analyze the pH of the samples at 25°C. The protein concentration of SG and SBB was determined by the Kjeldahl method (nitrogen conversion factor is 6.25) (Tian et al., 2010). The dry matter (DM), fat, and ash content of SG and SBB (around 1 g or 10 mL for SG or SBB samples, respectively) were determined according to the method described by AOAC (2000). Carbohydrate content was calculated by difference. The energy values of SG and SBB were calculated using Equation (1).

(1) $$\begin{array}{c} \text{Energy value}\;\left(\text{kcal}\;\mathrm{per}\;100\;g\;\text{or}\;100\;\mathrm{mL}\right)\\ =\left(\text{Protein}\%\times 4\right)+\left(\text{Carbohydrates}\%\times 4\right)+\left(\mathrm{Fat}\%\times 9\right) \end{array}$$

### 2.6 Calculation of SBB yield

The extraction yield of SBB was calculated using Equation (2):

(2) $$\mathrm{SBB}\;\text{yield}\;\left(\%\right)=\left(W1/\text{W2}\right)\times 100$$

where W1 and W2 are the weights of the extracted SBB and the sorghum grain (100 g), respectively.

### 2.7 Determination of starch and sugar content

The starch and sugar contents of the samples were determined by diluting the milled sorghum grain (SG) and SBB with water (1:50 w/v) and homogenizing them (Heidolph Instruments GmbH and CoKG, Germany) at 12000 rpm for 1 min. The starch level of the SG and SBB was determined by the method described in a previous study (Babolanimogadam et al., 2023).

Also, the reducing sugar content (RS) of SG and SBB samples was determined by the dinitrosalicylic acid (DNS) assay method described in a previous study (Miller, 1959). Firstly, the DNS reagent was prepared by dissolving 1.5 g of DNS in 30 mL of a 2 M NaOH solution. Concurrently, 45 g of sodium potassium tartrate was dissolved in 75 mL of distilled water. The two solutions were then combined, and the total volume was brought up to 150 mL with additional distilled water. For the assay, 1 mL of each diluted sample was mixed with an equal volume of the freshly prepared DNS reagent. The reaction mixture was incubated for 15 min in a boiling water bath, followed by cooling to 25°C. The volume of the cooled mixture was adjusted to 10 mL with distilled water. The optical density (OD) of the resulting solutions was measured at a wavelength of 540 nm. A calibration curve was constructed using glucose standards ranging from 0.0025% to 0.1%, and the RS content was quantified and expressed as a percentage.

### 2.8 Extraction of bioactive compounds

The previously described method with slight modifications was used for extracting bioactive compounds from milled SG and SBB samples (Awika et al., 2005). The SG and SBB (1 g or 1 mL, respectively) were diluted in acidified methanol with HCl (methanol:water:HCl 80:19.9:0.1 v/v) and homogenized at 12000 rpm for 2 min, and then agitated for 16 h at 25°C. The supernatants from all samples were collected after centrifugation at 5000 rpm for 15 min at 4°C. These extracts were used to determine the total phenolic content (TPC) and the antioxidant activity (AOA).

### 2.9 Determination of total phenolic content

The TPC was determined by the Folin–Ciocalteu method (Best et al., 2020). Briefly, 100 μL of the extracts were mixed with 750 μL of Folin–Ciocalteu reagent (0.2 N) and incubated for 5 min at 25°C. Then, 750 μL of sodium carbonate (7.5%) was added and incubated in a water bath at 40°C for 30 min. The absorbance was recorded at 725 nm, and the results were expressed in mg of gallic acid equivalents (GAE) per kg or L of the sample by using a standard curve prepared with different concentrations of gallic acid.

### 2.10 Evaluation of antioxidant activity

The AOA was assessed using the 1,1‐diphenyl‐2‐picrylhydrazyl (DPPH) assay (Sigma Aldrich Corp., St. Louis, MO, USA). Briefly, 50 μL of the extracts were mixed with 950 μL of DPPH solution (65 μmol/L in methanol) and incubated for 10 min at 25°C. The absorbance of the samples (As) was measured at 515 nm using a microplate reader. A blank (Ab) was prepared with a DPPH solution without the extract (Best et al., 2020).The DPPH scavenging activity (%) was calculated by Equation (3):

(3) $$\text{DPPH scavenging activity}\;\left(\%\right)=\left(\left(\mathrm{Ab}-\mathrm{As}\right)/\mathrm{Ab}\right)\times 100$$

### 2.11 Sensory evaluation

The sensory attributes of color, aroma, flavor, and overall acceptability of nine different SBB samples were evaluated by 15 trained panelists, including students and staff of the Ardabil University of Medical Sciences (aged between 19 and 40 years) using a 9‐point hedonic scale (1 = dislike extremely, 2 = dislike very much, 3 = dislike moderately, 4 = dislike slightly, 5 = neither like nor dislike, 6 = like slightly, 7 = like moderately, 8 = like very much, and 9 = like extremely). The samples were adjusted to 25°C and pH 6.7 and served in clear plastic cups containing 25 mL of the product with a random 3‐digit code at 25°C under normal daylight conditions. Filtered water was provided for the panelists to rinse their mouths between samples to minimize residual effects. Informed consent was obtained from all panelists.

### 2.12 Statistical analysis

All analyses of SBB treatments (*n* = 9) were performed in triplicate (*n* = 3), and results were expressed as means ± standard deviations. Data were statistically analyzed by one‐way analysis of variance (ANOVA) using SPSS for Windows (Version 22.0, SPSS Inc., Chicago, IL, USA). Duncan's test was applied to determine significant differences (*p* < .05). The sensory evaluation was analyzed by the non‐parametric Kruskal–Wallis test and continued by paired comparison using the Mann–Whitney *U*‐test.

## 3 RESULTS AND DISCUSSION

In this research, the effect of single and combination germination, acid, and α‐amylase treatments on the nutritional and functional properties of SBB, including SBB extraction yield, protein, ash, fat, carbohydrates contents, energy value, starch and reducing sugar content, total phenolic content, antioxidant activity, and also sensory properties of SBB were studied. The components of the SG are shown in Table 1. The results of this study indicated that the protein, fat, total carbohydrate, dry matter (DM), ash, and energy content of the SG were 12.82%, 3.37%, 76.57%, 94.56%, 1.8%, and 387.89 kcal/100 g, respectively. These findings were in agreement with the previous report by Ratnavathi and Komala (2016), who reported the protein (7.18 to 18.93%), fat (1.94 to 4.27%), and starch (30 to 80.75%) contents of sorghum grains. Juliano (1999) also reported the composition of sorghum seeds to include protein 8.3%, fat 6.3%, ash 2.6%, crude fiber 13.8%, and starch 50% in terms of g/100 g. The nutritional composition of sorghum grains depends on several factors, such as the variety of the grain, the growing location and weather conditions, the water and fertilizer use, and the processing methods (Kardeş et al., 2021; Lin et al., 2021; Tanwar et al., 2023).

**TABLE 1:** The chemical composition of whole sorghum grain (mean ± SD).

Component of whole sorghum grain	
DM (%)	94.56 ± 0.19
Total carbohydrates (%)	76.57 ± 0.26
Protein (%)	12.82 ± 0.30
Fat (%)	3.37 ± 0.09
Ash (%)	1.80 ± 0.10
Starch (%)	67.31 ± 0.12
Total fiber (%)	6.94 ± 0.14
Energy (kcal/100 g)	387.89 ± 0.15

The chemical composition of SBB is shown in Tables 2 and 3. The results of this study showed that the SBB yield was affected by the investigated treatments, with the lowest efficiency value being related to the control treatment (CT) with an efficiency of 79.3% and the highest being related to the acid‐enzymatic treatment (AET) with an efficiency of 90.27% (*p* < .05). The use of enzymes increased the SBB yield of all treatments compared to the CT treatment, which was consistent with the results of a previous study (Aiyer, 2005), which used enzymes for the digestion of starch and reduction of viscosity, increasing efficiency, and facilitating filtration.

**TABLE 2:** The chemical composition of SBB produced by different treatments (mean ± SD).

Treatments	DM (%)	Protein (%)	Fat (%)	Ash (%)	Carbohydrates (%)
CT	2.68 ± 0.38 ^a^ †	0.83 ± 0.00^e^	0.183 ± 0.006^a^	0.238 ± 0.006^a^	1.43 ± 0.38^b^
AT	5.46 ± 0.45^b^	0.67 ± 0.004^a^	1.388 ± 0.054^f^	0.948 ± 0.021^e^	2.46 ± 0.50^c^
En	2.53 ± 0.21^a^	0.82 ± 0.010^e^	0.433 ± 0.013^b^	0.405 ± 0.002^c^	0.87 ± 0.21^ab^
GT	2.87 ± 0.20^a^	0.98 ± 0.002^f^	1.186 ± 0.029^d^	0.257 ± 0.011^b^	0.45 ± 0.22^a^
GAT	5.98 ± 0.24^b^	0.74 ± 0.007^c^	1.277 ± 0.017^e^	1.068 ± 0.004^f^	2.90 ± 0.23^c^
GET	3.09 ± 0.42^a^	0.75 ± 0.008^d^	0.481 ± 0.020^c^	0.457 ± 0.004^d^	1.40 ± 0.42^b^
AET	6.94 ± 0.19^c^	0.72 ± 0.001^b^	0.151 ± 0.002^a^	1.236 ± 0.011^g^	4.84 ± 0.20^d^

*Note*: Values in each column followed by the different letters are significant at the 5% level.

Abbreviations: AET, acidic‐enzyme; AT, acid; CT, control; En, enzyme; GAT, germination‐acidic; GET, germination‐enzymatic; GT, germination.

^†^ Treatments.

**TABLE 3:** Other properties of SBB produced by different treatments (mean ± SD).

Treatments	pH	SBB yield	Starch (%)	Reducing sugar	Energy value (kcal/100 mL)
CT	6.39 ± 0.13	79.30 ± 0.36^a^ †	2.495 ± 0.040^f^	0.13 ± 0.01^a^	10.69 ± 1.53^a^
AT	2.14 ± 0.11	85.20 ± 2.45^b^	1.566 ± 0.012^e^	0.82 ± 0.04^b^	24.99 ± 1.76^cd^
En	5.79 ± 0.15	85.89 ± 3.47^b^	0.349 ± 0.007^b^	1.03 ± 0.02^d^	10.68 ± 0.86^a^
GT	5.45 ± 0.15	88.30 ± 0.99^bc^	0.455 ± 0.007^d^	0.94 ± 0.02^c^	16.39 ± 0.72^b^
GAT	2.12 ± 0.09	85.41 ± 0.80^b^	0.406 ± 0.004^c^	1.32 ± 0.03^e^	26.02 ± 0.97^d^
GET	5.38 ± 0.20	87.75 ± 1.26^bc^	0.331 ± 0.002^b^	1.59 ± 0.04^f^	12.94 ± 1.71^a^
AET	5.45 ± 0.53	90.27 ± 1.07^c^	0.072 ± 0.001^a^	3.40 ± 0.04^g^	23.57 ± 0.78^c^

*Note*: Values in each column followed by the different letters are significant at the 5% level.

Abbreviations: AET, acidic‐enzyme; AT, acid; CT, control; En, enzyme; GAT, germination‐acidic; GET, germination‐enzymatic; GT, germination.

^†^ Treatments.

The initial pH of different treatments of SBB depended on the type of treatment. The initial pH values of CT, acid treatment (AT), enzymatic treatment (En), AET, germination treatment (GT), germination‐acid treatment (GAT), and germination‐enzymatic treatment (GET) of SBB were 6.39, 2.14, 5.79, 5.45, 5.45, 2.12, and 5.38, respectively. The pH values of all treatments were adjusted to 6.7 before the next steps. In the case of the AET, the pH was also adjusted to 6.7 before the second‐stage treatment.

The SBB yield of germinated treatments was higher than the others, which was in line with the research results of previous studies (Hübner & Arendt, 2013; Nkhata et al., 2018), who reported that the alpha‐amylase enzyme was produced during the germination process and the amount of grain starch decreased. Moreover, during germination, the soluble fiber content decreased due to the high water‐binding capacity. The germination treatment, along with the alpha‐amylase enzyme, led to an increase in yield. These results were in accordance with the research of the previous study (Ba et al., 2013). They concluded that the amount of total solids increased with the action of amylases on starch and the generation of maltodextrin. This action resulted in an increase in total solids due to the action of amylases on starch and the production of maltodextrin (De Mesa‐Stonestreet et al., 2010; Sadaf et al., 2023). Generally, the yield of SBB increased by using various treatments, such as reducing the particle size, applying heat, using acid hydrolysis, applying enzymatic methods, and performing germination. Enzyme treatment alone or in combination with other methods was one of the potential actions that led to an increase in yield along with an increase in the quality and acceptability of plant‐based beverages, and this yield was a function of the amount of alpha‐amylase enzyme used. Also, the effect of enzyme treatment was time‐dependent, and as time increased, the yield also increased (Choi et al., 2010). It was observed that the efficiency values increased with increasing time, and after complete digestion of starch, other components and structures, such as fibers and aleurone cell walls, decreased the efficiency.

The results showed that the AET had the highest dry matter (DM) content (6.94%), compared to other treatments (*p* < .05), followed by other acidic treatments, which might be due to the phosphoric acid content of these treatments. Moreover, the AET treatment of SBB had the lowest starch content (0.072%), followed by other enzymatic treatments. The results indicated that all treatments had starch digestibility properties, but the combined treatments were more effective than single treatments in reducing the starch content, which might be due to the sufficient digestion time and more digestion steps. The reduction in starch content of sorghum during germination was also reported by a previous study (Oseguera‐Toledo et al., 2020). Starch is the main component of sorghum grain, which may cause different technological problems during treatments due to the gelatinization during thermal treatments (Hill et al., 2012). It was shown that more digestion of starch led to a higher extraction yield of components (Aiyer, 2005).

The RS content of control treatment (CT), acid treatment (AT), enzymatic treatment (En), AET, germination treatment (GT), germination‐acid treatment (GAT), and germination‐enzymatic treatment (GET) samples was 0.13%, 0.82%, 1.03%, 3.4%, 0.94%, 1.32%, and 1.59%, respectively. RS was at its highest level in AET, GET, GAT, and GT treatments, and the lowest in CT. The use of acid and enzyme, as well as germination, or the use of acid and enzyme along with germination, had an increasing effect on the RS content of SBB. These results were consistent with the observations of Li et al. (2016), who reported that acid treatment increased the amount of glucose resulting from the decomposition of lignocellulosic materials in the environment. The results were also in agreement with the results of a previous study (Nair et al., 2018), which reported that acid pretreatment using diluted phosphoric acid caused the decomposition of plant cellulosic materials and ultimately increased the content of fermentable sugars.

The results of this study showed that the highest amount of protein was related to GT, with a value of 0.98%, which was significantly (*p* < .05) higher than other treatments. The amount of protein in AT was at its lowest, with a value of 0.67%, and was significantly (*p* < .05) lower than other treatments. This factor might be due to the release of seed proteins from complex plant tissues, the presence of free peptides as a result of germination, and the application of heat during the preparation stage of the treatments. These results were consistent with the findings of a previous study (Arouna et al., 2020). Also, GT and AT had significantly (*p* < .05) the highest and lowest protein yield values (67.55% and 44.42%), respectively. These results were consistent with the findings of a previous study (Wang et al., 1999), which reported that protein extraction in alkaline conditions was higher than in acidic conditions due to changes in the ionic or surface properties of proteins.

The results showed that the fat content of AT (1.38%) was significantly (*p* < .05) higher than other treatments, followed by GAT and GT (1.27% and 1.18%, respectively). In general, AT treatments had a higher fat content than the other treatments, which was consistent with the research conducted by previous studies (Palmquist & Jenkins, 2003; Zou et al., 1999), who reported that the acid hydrolysis of grains led to a higher extraction of their fat, and this method was suitable for extracting fat. Similarly, a previous study reported that acid hydrolysis leads to the release of bound fats and causes their further extraction (Strange & Schaich, 2000).

This study also observed that the germination treatment (GT) increased the fat content compared to the non‐germinated treatments, which was consistent with the results of a previous study (Saithalavi et al., 2021), who reported that the fat content of sorghum flour increased after sprouting. Moreover, the acid‐enzymatic treatment (AET), the germination‐acid treatment (GAT), and the acid treatment (AT) had the highest amount of ash with 1.23%, 1.06%, and 0.94%, respectively, which were significantly (*p* < .05) different from other treatments; the differences might be due to the addition of phosphoric acid during the preparation process. The results showed that the energy values of GAT and AT of the SBB treatments were higher than those of other treatments. The findings indicated that the energy value of the treatments was contingent upon their dry matter (DM) content.

The TPC and AOA values of SBB are shown in Table 4. Among the SBB treatments, the highest phenolic compounds were observed in the AET with 44.56 mg/L, which was significantly (*p* < .05) higher than other treatments. This factor might be caused by the breakdown of protein materials and types of carbohydrates in sorghum seeds, which were broken down by acid and enzyme, respectively, and phenolic materials were not able to combine and blend with proteins and carbohydrates due to the application of temperature (Barros et al., 2020). GT treatment had the second highest TPC value with 37.59 mg/L, which might be attributed to the increase in enzyme activities and decomposition of components containing phenolic compounds due to germination. The antioxidant activity (AOA) value of enzymatic treatment (En), GT, and germination‐enzymatic treatment (GET) treatments was higher than that of AT, GAT, and AET treatments, which might be due to the role of acid in denaturing antioxidant compounds (Adebo & Gabriela Medina‐Meza, 2020). The results indicated a tenuous relationship between TPC and AOA in the context of SBB. The results showed that the TPC of the GET increased by 11.55% compared to the GAT, and the AOA of the GET increased by 99.13% compared to the GAT. Also, while the TPC of AET increased by 64.85% and 170.22% compared to the AT and En treatments, respectively, its AOA increased by 17.10% and decreased by 45.14% compared to the AT and En treatments, respectively. There are many reasons why there is an ambiguous relationship between the TPC and AOA, such as nonphenolic compounds with antioxidant activity such as ascorbic acids, tocopherol, and β‐glucan oligosaccharides, the complex interactions among antioxidant compounds, the variability inherent in assay techniques (Sun et al., 2020), and also the different antioxidant efficacies of phenolic compounds that showed low antioxidant properties (Brand‐Williams et al., 1995).

**TABLE 4:** TPC and AOA content of SBB produced by different treatments (mean ± SD).

Treatments	Total phenolic content (mg/L)	Antioxidant properties (mg BHT eq./L)
CT	28.14 ± 0.52^d^ †	142.85 ± 21.00^b^
AT	27.03 ± 0.34^c^	63.03 ± 18.83^a^
En	16.49 ± 0.24^a^	134.55 ± 25.82^b^
GT	37.59 ± 0.25^e^	135.77 ± 25.51^b^
GAT	25.46 ± 0.26^b^	59.76 ± 7.55^a^
GET	28.40 ± 0.37^d^	119.00 ± 23.12^b^
AET	44.56 ± 0.14^f^	73.81 ± 7.85^a^

*Note*: Values in each column followed by the different letters are significant at the 5% level.

Abbreviations: AET, acidic‐enzyme; AT, acid; CT, control; En, enzyme; GAT, germination‐acidic; GET, germination‐enzymatic; GT, germination.

^†^ Treatments.

The sensory evaluation of SBB treatments is shown in Table 5. The results showed that the color of SBB prepared by different treatments varied from 6.27 for CT to 8.7 for En. In the evaluations of the sensory characteristics of color, aroma, and flavor, all the treatments evaluated by SBB were higher (*p* < .05) than the CT. But in the aroma evaluations, AT, En, and AET were (*p* < .05) lower than CT and higher in GT, GAT, and GET. (*p* < .05) were more than the CT sample. The aroma points of AT, En, and AET are (*p* < .05) lower than CT and higher in the GT, GAT, and GET samples. Also, overall acceptability of all the SBB treatments was higher than that of the CT sample. The results showed that all sensory evaluation scores of En, GT, and GET treatments were above 8. This is the primary score for consumer acceptability and marketability. Also, the overall acceptance score of En, AT, GAT, GT, and GET treatments was 7.73, 7.5, 8.03, 8.43, and 8.77. The En treatment showed the highest color score (8.7), GET showed the highest aroma score (8.67) and overall acceptability (8.77), and GAT (8.6) showed the highest flavor score.

**TABLE 5:** The sensory evaluations of SBB produced by different treatments (mean ± SD).

Treatments	Color	Aroma	Flavor	Overall acceptability
CT	6.27 ± 0.15	6.67 ± 0.15	7.17 ± 0.15	6.17 ± 0.21
AT	6.60 ± 0.10	7.77 ± 0.15	6.83 ± 0.15	7.50 ± 0.20
En	8.70 ± 0.20	7.33 ± 0.21	6.23 ± 0.21	7.73 ± 0.15
GT	8.17 ± 0.20	8.13 ± 0.15	7.80 ± 0.10	8.43 ± 0.25
GAT	6.83 ± 0.06	7.17 ± 0.15	8.60 ± 0.10	8.03 ± 0.15
GET	7.83 ± 0.21	8.67 ± 0.15	8.17 ± 0.15	8.77 ± 0.15
AET	7.70 ± 0.20	7.17 ± 0.15	6.13 ± 0.15	6.53 ± 0.20

## 4 CONCLUSION

This study investigated the effect of different treatments on the nutritional and functional properties of sorghum bran beverage (SBB). The results revealed that the use of different treatments affected the different characteristics of SBB. The highest protein and fat contents in sorghum‐based beverages (SBB) were attained through germination (GT) and acidic (AT) treatments, yielding 0.98% and 1.388%, respectively. Additionally, the acid‐enzyme treatment (AET) exhibited the highest carbohydrate content at 4.84%. All treatments resulted in a greater beverage yield when contrasted with the control, with AET achieving the highest SBB yield at 90.27%. The greatest energy value for SBB, 26.02 kcal/100 mL, was recorded for the germination‐acidic treatment (GAT). The employment of phosphoric acid and α‐amylase enzyme (AET) alongside the germination (GT) of sorghum grains led to an increase in phenolic compounds in SBB by 58.35% and 33.58%, respectively, compared to the control. Moreover, all treatments resulted in a reduction in antioxidant activity relative to the control. Notably, the use of phosphoric acid had the most pronounced diminishing effect on the antioxidant activity (AOA) of GAT, AT, and AET, with decreases of 58.17%, 55.88%, and 48.33%, respectively, when compared to the control treatments. Moreover, the acid, enzyme, and sprouting treatments of the diluted sorghum seeds, either alone or in combination, were more effective on the studied factors and enhanced the acceptance characteristics of the beverage compared to the control sample. In general, using different treatments that caused high digestion of the wall structure of sorghum grain contributed to the higher nutritional and functional properties of this plant‐based beverage.

## AUTHOR CONTRIBUTIONS

**Seyed Hasan Sajjadi Alhashem:** Conceptualization (equal); formal analysis (equal); investigation (equal); methodology (equal); software (equal); writing – original draft (equal). **Mohammad Reza Ehsani:** Conceptualization (equal); formal analysis (equal); methodology (equal); supervision (equal); validation (equal); writing – review and editing (equal). **Afshin Akhondzadeh Basti:** Conceptualization (equal); software (equal); supervision (equal); writing – review and editing (equal). **Anoosheh Sharifan:** Data curation (equal); supervision (equal); writing – review and editing (equal).

## FUNDING INFORMATION

This research was conducted without external funding.

## CONFLICT OF INTEREST STATEMENT

The authors confirm that they have no conflicts of interest with respect to the work described in this manuscript.

## ETHICAL STATEMENTS

All panelists enrolled in this study provided written informed consent. Also they were informed that they can withdraw from the evaluation at any time without giving a reason.

## Data Availability

There is no primary data associated with this manuscript.
